# Cost Assessment of Five Different Maize Grain Handling Techniques to Reduce Postharvest Losses from Insect Contamination

**DOI:** 10.3390/insects11010050

**Published:** 2020-01-10

**Authors:** Bernard Darfour, Kurt A. Rosentrater

**Affiliations:** 1 Radiation Technology Centre, Biotechnology and Nuclear Agriculture Research Institute, Ghana Atomic Energy Commission, P.O. Box LG 80 Legon-Accra, Ghana; bdarfour@iastate.edu; 2 Agricultural and Biosystems Engineering Department, Elings Hall, Iowa State University of Science and Technology, 3327 Elings Hall, Ames, Iowa, IA 50011, USA

**Keywords:** cost analysis, maize grain, silo, PICS bag, phosphine, polypropylene bag

## Abstract

Farmers in developing nations encounter high postharvest losses mainly attributable to the lack of modern techniques for threshing, cleaning, grading, and grain storage. Mechanized handling of grain in developing countries is rare, although the technology is effective against insects and pest infestations. The objective was to evaluate the cost-effectiveness of five grain handling techniques that have the ability to reduce postharvest losses from insect infestation. The five methods were metal silo plus all accessories (m. silo + acc.), metal silo only (m. silo), woven polypropylene plus phosphine (w. PP. + Phos.), woven polypropylene only (w. PP.), and Purdue Improved Crop Storage bags only (PICS). The functional unit used was handling 1 kg of maize grain. The cost analysis of each technique was calculated based on equations using a spreadsheet. The annual capital and operational costs of handling using m. silo + acc. or m. silo were very high, unlike the PICS, w. PP. + Phos., or w. PP. The annual capital and operational costs decreased as production scale increased. Food security (due to reduced insects and pest infestations) and financial prospects of farmers can improve when the grain is mechanically handled with m. silo + acc. or m. silo.

## 1. Introduction

Farmers in Ghana and other sub-Saharan African (SSA) nations encounter high postharvest losses mainly attributable to the lack of modern techniques for threshing, cleaning, grading, and grain storage. Due to this, quantitative losses after harvest are experienced in the field (15%) and during processing (13% to 20%) and storage (15% to 25%) [1]. In support, the FAO [2] indicated that 20%–30% of cereals are lost after harvest in most developing countries. Other studies [3,4] have reported maize grain damage even up to 80%. Many farmers sell almost all their harvested grain a few weeks after harvest because they are constrained by income, debt, or lack of proper storage facilities [5].

**Figure 1:**
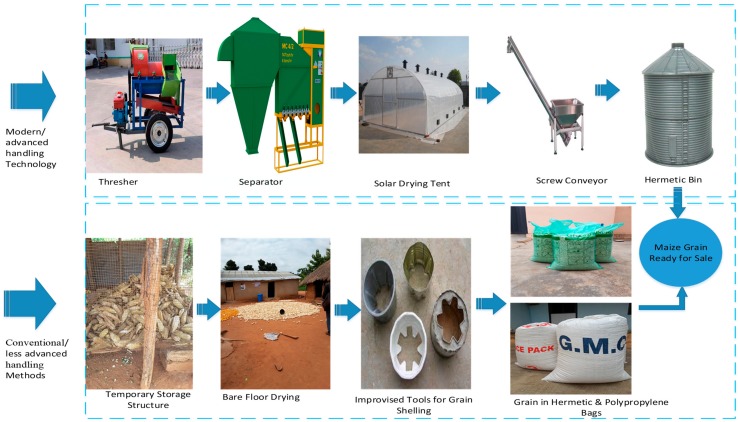
System boundary for the cost analysis of various maize handling techniques.

Farmers can maintain or improve their grain quality and quantity if modern techniques of handling grain are adopted. The modern techniques for handling grain include the use of mechanical threshers for grain shelling, gravity separators (to remove stones and other foreign materials) for cleaning and grading, solar tents for drying the grain, conveyors for the convenient conveyance of grain, and the use of airtight metal or steel storage bins. The setup for the five techniques is found in Figure 1. All these equipment or materials are used to minimize insects and pest infestations, and to maintain the grain quality. Grain quality and quantity can be maintained so that farmers can gain substantial profits if grain price rises. Efforts have been made to introduce some of the relatively inexpensive modern techniques to farmers in Africa. Such techniques include the use of hermetic containers and bags (jars or silos or Purdue Improved Crop Storage (PICS) bags or GrainPro bags (SuperGrainbag Premium RZ) which limit or prevent the exchange of oxygen and carbon dioxide between the external environment and the stored product), locally made hermetic metal silos, and insecticides (e.g., Actellic Super (Syngenta Actellic Gold Dust) and phosphine (Phostoxin)). A combination of two or more of such methods had been studied and introduced to farmers in SSA to reduce grain damage due to insects, pests, and molds [3,6,7,8,9]. In developing nations, handling of grain using entirely mechanized technology from threshing to storage is rare. Although mechanized technology is expensive, its efficacy in keeping grain safe during storage is high.

To surmount any financial obstacles associated with the mechanized mean of grain handling, farmers can form co-operatives at local levels. This gives them a common voice as a first step to apply for financial credit. Individual farmers face a difficult task in an attempt to obtain financial credit from financial organizations in SSA, and Ghanaian farmers are not exempted. Nevertheless, the process becomes less cumbersome in the name of farmer co-operatives. This study aimed to establish the capital cost, operational cost, and profit associated with five grain handling techniques. Therefore, the study aimed at evaluating the cost-effectiveness of five grain handling techniques that have the ability to reduce postharvest losses from insect infestation.

## 2. Materials and Methods

The cost assessment of five grain handling techniques (methods) was computed in an MS spreadsheet. The five methods were metal silo plus all accessories (m. silo + acc.), metal silo only (m. silo), woven polypropylene plus phosphine (w. PP. + Phos.), woven polypropylene only (w. PP.), and PICS bags only (PICS). All accessories comprise of a thresher, gravity separator, screw conveyor, and HDPE + UV solar tent dryer.

### 2.1. Goal and Scope of the Study

The study was to evaluate the cost-effectiveness of handling maize grain in Ghana using five storage techniques (Table 1). Based on the results, farmers could decide to either (or not) form co-operatives to enhance their food security and income security. It could also be useful to the government of Ghana in the implementation of some agricultural policies.

### 2.2. System Boundary

The costs incurred by farmers during and after cultivation, and at pre-harvest and harvest, were all excluded from the system boundary. The cost incurred during the transport of grain from farm to home (handling site) was also excluded. The system boundary for the cost assessment considered only the portions that Figure 1 highlights. The considered losses were threshing to storage only.

### 2.3. Functional Unit (FU)

The FU of this study was handling 1 kg of maize grain.

### 2.4. Assumptions

- Harvested grain was brought home (handling site) for temporary (about a week) storage before threshing.
- The inflation rate and changes in the future exchange rate (cedi and dollar) in Ghana were not considered in the calculation.
- New equipment bought from China and the shipping costs were included in the analysis.
- The initial moisture content (MC) (wet basis) of grain at harvest was 20%, then dried down to 13%, and 13% MC was used in the calculations. Other assumptions are also found in Table 2 and Table 3.

### 2.5. Experimental Design

The independent variables and scenarios that were simulated can be found in Table 1. The equations from Table 4 were then utilized to conduct the cost assessments. Equation (13) was used to calculate the annual capital cost in all 240 scenarios that were studied. The annual operational cost was calculated as the sum of Equations (19) and (24), the yearly profit was calculated with Equation (25), the total annual benefit was calculated with Equation (12), and the breakeven point was calculated using Equation (27). All the equations used in the calculations can be found in Table 4, and outputs of the calculations were subsequently graphically illustrated.

## 3. Results and Discussion

### 3.1. Annualized Capital and Operational Costs, Benefits, and Profits

**Figure 2:**
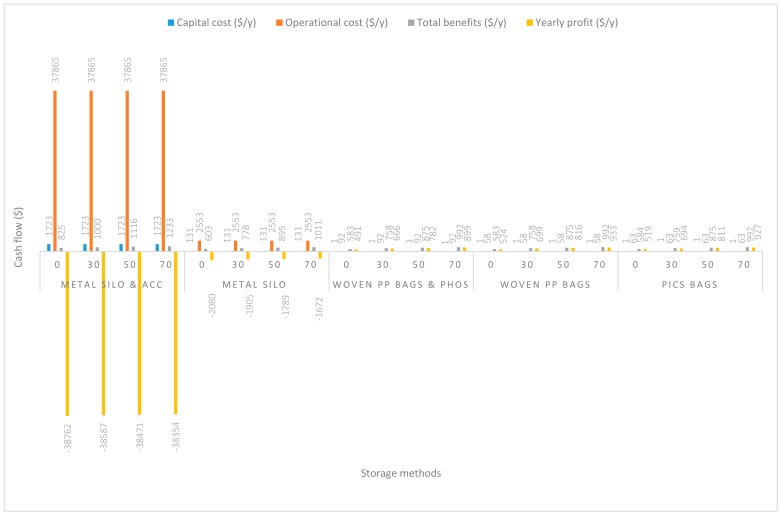
Annual capital and operational costs (US dollars), total annual benefits, and profits (US dollars) for each handling/storage method at 2.8 Mg/y handling capacity for 0%, 30%, 50%, and 70% grain price increase.

In Figure 2, the grain was handled at a capacity of 2.8 Mg/y. The capital and operational costs of using metal silo plus all accessories (m. silo + acc.) during grain handling were extremely high, $1722.55/y and $37,864.57/y respectively. About $130.85/y and $2552.54/y were recorded respectively as the capital and operational costs of using metal silo only (m. silo). Based on the usage of PICS bags only (PICS) a substantial reduction was observed in both the capital and operational costs, which were $1.12/y and $63.27/y, respectively. A similar lower-cost trend was recorded using woven polypropylene plus phosphine (w. PP. + Phos.) and woven polypropylene only (w. PP.). The capital and operational costs of handling grain with w. PP. + Phos. were $1.05/y and $91.92/y, and capital and operational costs of handling grain with w. PP. were $0.63/y and $58.32/y, respectively. Yearly profits margin ($/y) recorded handling grain with m. silo + acc. were negative even when the price of maize was increased to 70% (−$38,353.98/y). Grain handling using m. silo recorded a yearly negative profit at even a 70% increase in the price of maize (−$1672.09/y). However, handling grain using PICS or w. PP. + Phos. or w. PP. recorded positive yearly profits. The yearly profits increased with the estimated increases in maize price. At no price increase, w. PP. recorded the highest profit of $524.48/y, and was followed closely by PICS ($519.11/y) and w. PP. + Phos. ($490.52/y).

**Figure 3:**
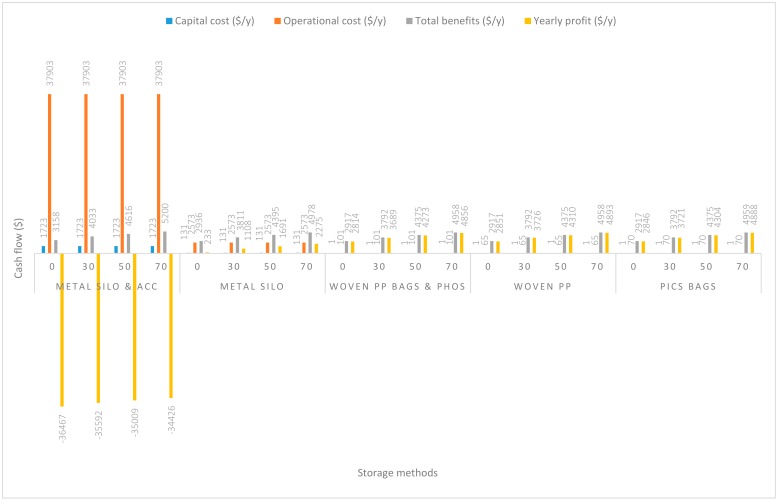
Annual capital and operational costs (US dollars), total annual benefits and profits (US dollars) for each handling/storage method at 14 Mg/y handling capacity for 0%, 30%, 50%, and 70% grain price increase.

In Figure 3, the handling capacity was increased to 14 Mg/y. The yearly capital costs remained the same as in 2.8 Mg/y for all five handling methods. Nevertheless, increases in yearly operational costs were recorded. Handling of grain using m. silo + acc., m. silo, w. PP. + Phos., PICS, and w. PP. respectively recorded increases of $38.19/y, $19.99/y, $9.58/y, $6.38/y, and $6.38/y above the operational costs incurred in 2.8 Mg/y. Except for the handling of grain in the m. silo + acc. scenario, profits for all other scenarios were positive, even without any estimated maize price increase. Importantly, the reduction in the negative yearly profit by $2295.14/y using m. silo + acc. should be acknowledged. The yearly increase in profits (at no grain price increase) recorded above the 2.8 Mg/y handling capacity were $2313.35/y, $2326.95/y, $2323.76/y, and $2326.95/y, respectively, for m. silo, PICS, w. PP. + Phos., and w. PP. The estimated increases in grain prices from 30% to 70% resulted in corresponding increases in profits according to a similar trend for all five handling methods.

**Figure 4:**
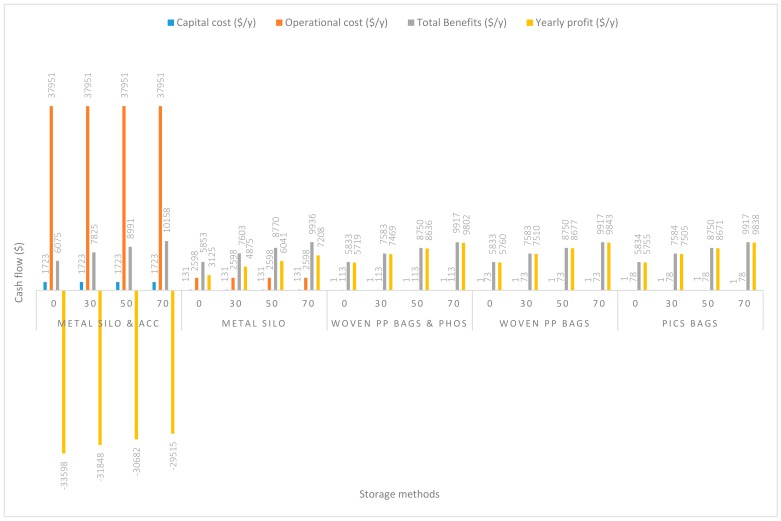
Annual capital and operational costs (US dollars), total annual benefits and profits (US dollars) for each handling/storage method at 28 Mg/y handling capacity for 0%, 30%, 50%, and 70% grain price increase.

In Figure 4, the handling capacity was increased to 28 Mg/y. Capital costs remained the same. There were slight increases in operational costs compared to that in the 14 Mg/y capacity. The additional increases in operational costs were $47.74/y, $24.99/y, $11.97/y, $7.98/y, and $7.98/y respectively associated with grain handling using m. silo + acc., m. silo, w. PP. + Phos., PICS, and w. PP. Yearly profits similarly increased and have been reported in decreasing order of w. PP. ($5760.11/y), PICS ($5754.75/y), w. PP. + Phos. ($5718.98/y), m. silo ($3124.59/y), and m. silo + acc. (−$33,598.24/y).

Based on the capital cost and operational cost analysis, handling maize grain with m. silo + acc. was very expensive compared to using m. silo. The total costs (sum of capital and operational costs) of handling grain using either m. silo + acc. or m. silo would be beyond the financial capability of any single individual smallholder farmer in a developing nation. Good storage systems demand huge capital and technical skills [23] which are impractical for smallholder farmers. However, the capital and operation costs of handling grain with PICS or w. PP. + Phos or w. PP. were within the financial capabilities of smallholder farmers. None of the three latter handling methods exceeded 0.08% of the capital costs and 0.3% of the operational costs of handling grain with m. silo + acc. Farmers would start making profits on their sales even at capacities as low as 2.8 Mg handling grain with PICS, w. PP. + Phos, and w. PP. The profits decreased from handling grains with w. PP., PICS to w. PP. + Phos. To overcome the financial challenges associated with handling grain using m. silo or m. silo + acc., farmers could come together to form farmer co-operatives. Farmer co-operatives help increase production and incomes of members through the possibility of accessing financial credits, agricultural inputs, information, and markets [24]. The co-operatives could engross the operational and capital costs, and this helps to have equity capital and debt capital to begin, and also to contract loans from financial institutions. According to the [25], equity capital refers to ownership capital provided by members in a co-operative, and the money that is borrowed is debt capital. The study by [26] indicated that agricultural co-operations fortify the power of smallholder farmers to bargain either for loans or good grain market prices. As reported in the [27], a maize-growing association in Ghana (Masara N’Arziki), started between 2008 and 2012, is now a mammoth association in West Africa with an estimated revenue of US$369.00 per farmer per hectare in 2012. Co-operatives could increase farmers’ handling capacity to make profits as quickly as possible. Alongside the financial benefits members also benefit from the improved product, service quality, reduced risks, and social and economic empowerment through the ability to partake in decision-making processes. Increased participation in Farmer Field School (FFS) in Kenya, Uganda, and Tanzania in 2010 resulted in improved crop productivity, production, and income [28]. The available marketing opportunities increase for storing grain beyond harvest. However, the choice of handling or storage method is highly dependent on the relative cost of the method and feasibility regarding the overall harvesting, handling, and marketing system [23].

### 3.2. The Annual Capital and Operational Costs, and Profits at Percentage Grain Losses

**Figure 5:**
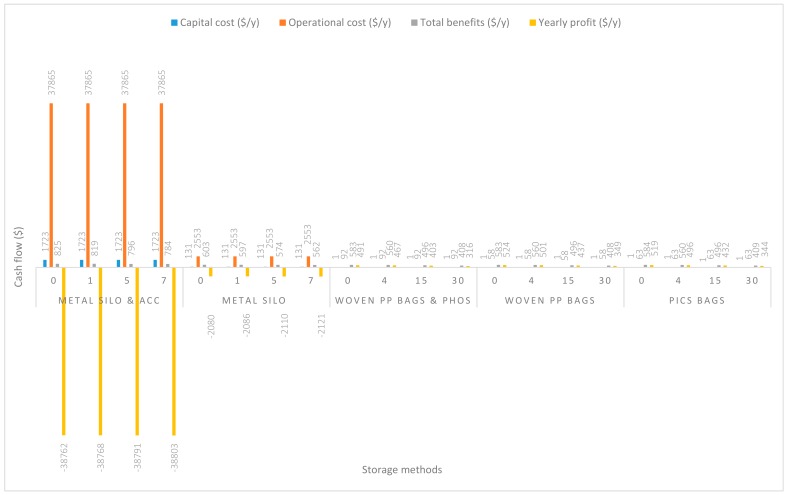
Annual capital and operational costs (US dollars), total annual benefits and profits (US dollars) for each handling/storage method at 2.8 Mg/y handling capacity for 0% to 7%, and 0% to 30% grain quantity losses.

**Figure 6:**
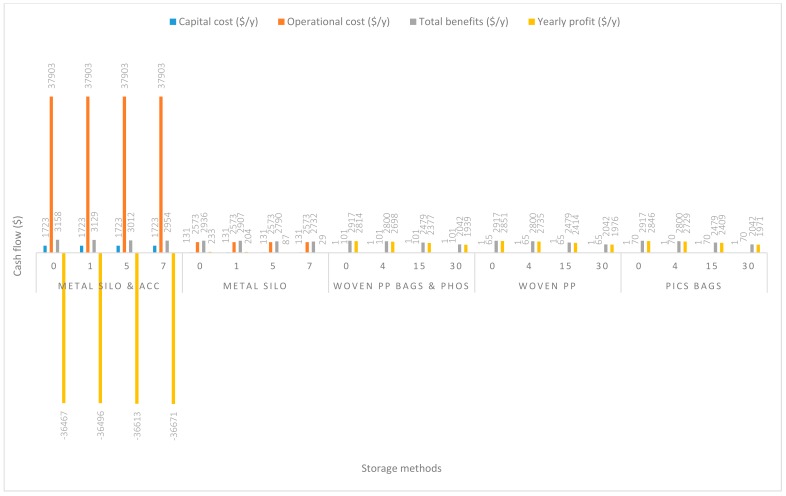
Annual capital and operational costs (US dollars), total annual benefits and profits (US dollars) for each handling/storage method at 14 Mg/y handling capacity for 0% to 7%, and 0% to 30% grain quantity losses.

**Figure 7:**
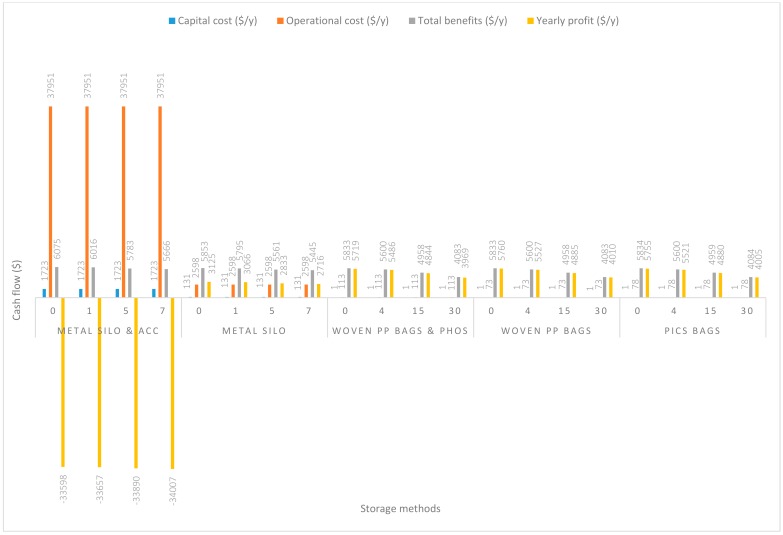
Annual capital and operational costs (US dollars), total annual benefits and profits (US dollars) for each handling/storage method at 28 Mg/y handling capacity for 0% to 7%, and 0% to 30% grain quantity losses.

In Figure 5, the capital and operation costs remained the same as were recorded in Figure 2 with regards to m. silo + acc., m. silo, w. PP. + Phos., w. PP., and PICS. The m. silo + acc. and m. silo upon subjection to losses in grain quantity of 0%, 1%, 5%, and 7%, the corresponding yearly profits were all in the negatives (although in increasing order of % reduction in quantity). However, the negative values ($) at zero percent loss in grain quantity were considerably improved. Similarly, yearly profits recorded by handling grain with w. PP. + Phos. or w. PP. or PICS, although positive, the profits reduced correspondingly with the increasing percentages of losses in grain quantity from 0%, 4%, 15%, to 30%. Figure 6 and Figure 7 respectively correspond to Figure 3 and Figure 4 concerning the capital and operational costs, which remained the same. Similar trends of negative profits were recorded when m. silo + acc was used, but much better because of the increase in handling capacity. The m. silo at this point recorded profits (positive) like that of w. PP. + Phos., w. PP., and PICS irrespective of the percentage loss in grain quantity.

Despite the expensive nature of handling grain with m. silo + acc., there are many significant advantages over the other methods, including maintaining grain quality and quantity with loses below 7%. Generally, grain elevators experience less than 1% loss during storage and handling, and about 1.4% normal shrinks [29,30], which are below the estimated percentage losses used in this study. Hermetic storage bins are more voluminous compared to self-built silos (locally made), and grain should have a safe MC before storage. The effectiveness of most locally built metal silos (m. silo) has been tested in many studies, and the experienced maize grain weight loss was minimal and relatively close to that of m. silo + acc. Negligible [12], 0.28% [8], 1.8% [3], and less than 7% [4] maize grain weight losses have been associated with using m. silo to store grain for at least 6 months.

Grain stored in PICS could experience grain losses between 4% and 12% [3,4,13]. Commonly, the percentage of grain weight loss was between 0% and 0.31% [6,8,9,13,31]. By contrast, the percentage of grain quantity loss encountered using w. PP. could be up to 33.8% [3], 17.95% [31], and 15% [9]. However, there could be some exceptions, such as [13] and [6], which revealed 2.4% and 0% losses, respectively. The efficacy of w. PP. + Phos. (or different insecticides) closely relates to PICS as between 0.03% and 12.3% losses normally occur, although 34% or greater have been reported [3,4,8,9,13]. Hermetic storage (bag or m. silo) has demonstrated superiority in preventing insect infestation and grain damage as well as losses in stored maize grain, although insecticides could be equally effective [4,8]. Nevertheless, the low operational costs and high yearly profits accrued using PICS surpass that of W. PP. + Phos. or w. PP. and, hence, are economically practicable for farmers that cannot purchase m. silo.

The m. silo protects grain against harsh climate, and infestation by insects, pests, and mold [23,32]. Comparatively, w. PP. + Phos., w. PP., and PICS are susceptible to harsh weather, insects, pests, and sometimes molds, and have relatively short lifecycles. PICS bags are more efficient in reducing grain losses compared to w. PP. under similar storage conditions [33,34]. However, hermetic bags could get damaged due to punctures from sharp end objects, or be abraded or perforated by rodents and insects [3,35]. Both w. PP. and PICS could burst during handling or transport and lose their usefulness, which subsequently results in an extra financial burden to farmers. Synthetic insecticides are expensive, maybe ineffective or adulterated or rare in the markets, and known to have detrimental health and environmental effects [36,37]. The results of this current assessment closely compare to earlier studies that used w. PP. + Phos., w. PP., and PICS regarding profit-making. However, using PICS has many advantages, including 3–4 years reusability, which results in reduced operational costs compared to using w. PP. + Phos. or w. PP. Jones et al. [9,38] reiterated the dramatic increase in profit when storage was extended to eight months in wait for a maximum price. In their study, the use of PICS and w. PP. + insecticide (Sofagrain) recorded positive returns on storage compared to w. PP. (without insecticides).

Worth noting was the high returns on PICS storage due to its reusability even though the capital cost was high compared to operation cost (low). Abass et al. [1] estimated that due to the lack of mechanized grain handling techniques in developing countries, grain quantitative losses of about 13% to 20%, and 15% to 25% happen during processing and storage, respectively. The huge postharvest losses experienced in developing countries are greatly attributable to the absence of good techniques to separate foreign particles and debris, and damaged or unhealthy grain (diseased grain or insect-infested grain or mold-infested grain) before storage. In spite of the costly capital and operational costs of handling grain using m. silo + acc. (mechanized), the fact that it could reduce losses in grain quantity makes this a preferred option. M. silo + acc. could maintain grain quality and quantity in storage, and could also contribute to improved food security and income of farmers. Manandhar et al. [39] further asserted that the use of w. PP. resulted in low quality and high loss in grain, w. PP. + Insecticide led to low grain quality and loss, while PICS ensured high grain quality and low loss. Reasonably, amidst the enormous benefits associated with using PICS, it had the lowest operational cost, and the capital cost was slightly high.

It would be appropriate for the government of Ghana to initiate the use of m. silo + acc. in grain handling. This is because of the government’s many agricultural flagship programs including planting for food and jobs, and planting for export and rural development.

### 3.3. Economies of Scale for Capital and Operational Costs

**Figure 8:**
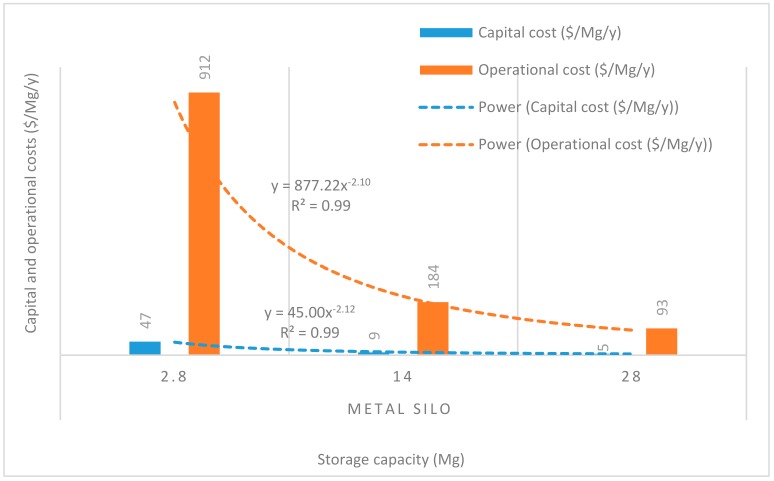
Economies of scale of capital and operational costs ($/Mg/y) of using metal silo only.

**Figure 9:**
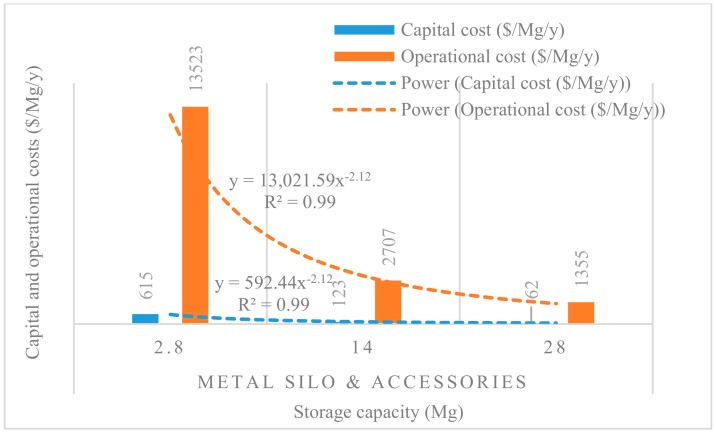
Economies of scale of capital and operational costs ($/Mg/y) of using metal silo and all accessories.

**Figure 10:**
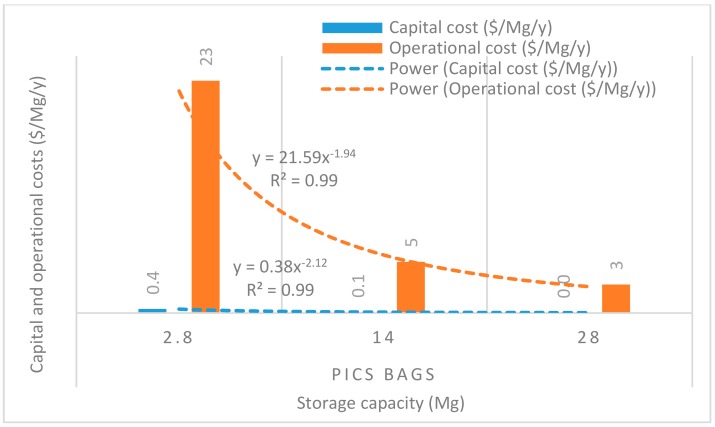
Economies of scale of capital and operational costs ($/Mg/y) of using PICS bags only.

**Figure 11:**
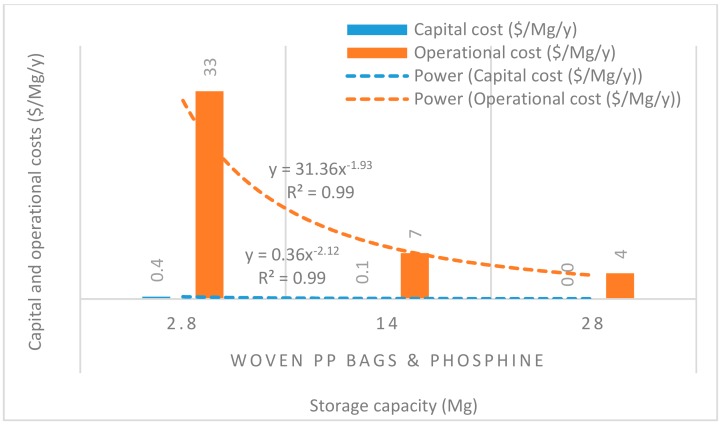
Economies of scale of capital and operational costs ($/Mg/y) of using woven polypropylene bags and phosphine tablets.

**Figure 12:**
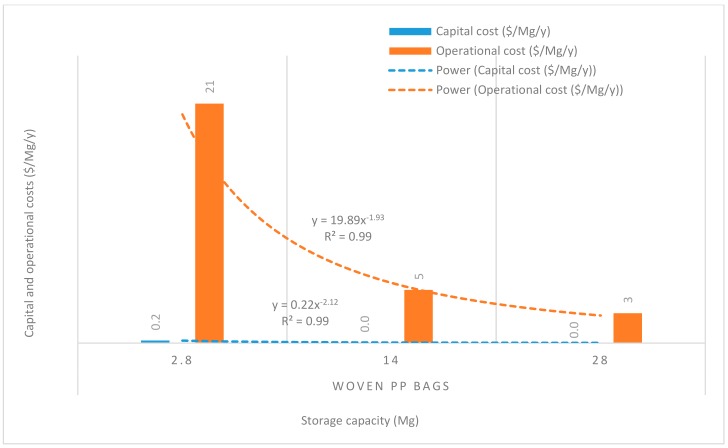
Economies of scale of capital and operational costs ($/Mg/y) of using woven polypropylene bags only.

Figure 8, Figure 9, Figure 10, Figure 11 and Figure 12 show the economies of scale of capital and operational costs of handling maize grain using the five handling methods (m. silo + acc., m. silo, w. PP. + Phos., w. PP., and PICS). As was anticipated, the capital and operational costs ($/Mg/y) decreased drastically with the increased handling capacity from 2.8 to 14 to 28 Mg. Among the five handling methods, the use of w. PP. recorded the lowest capital and operational costs at each handling capacity.

Grain handling with the m. silo had the capital and operational costs ($/Mg/y) less than 9% of that of m. silo + acc. across the 2.8 to 28 Mg capacity. The capital and operational costs required using PICS, w. PP. + Phos., and w. PP. respectively recorded less than 0.1% and 0.3% of using m. silo + acc. across the 2.8 to 28 Mg capacity. However, PICS, w. PP. + Phos., and w. PP. recorded less than 1% and 3% of the capital and operational costs of using m. silo across the 2.8 to 28 Mg capacity. Capital and operational costs of w. PP. + Phos. were about 36% higher than that of w. PP. across the 2.8 to 28 Mg capacity. Meanwhile, the capital cost of PICS was about 7% higher than that of w. PP. + Phos., and the operational cost of PICS was 31% less than that of w. PP + Phos. across the 2.8 M to 28 Mg capacity. The capital cost of PICS was about 44% higher than that of w. PP., and the operational cost of PICS was about 9% less than that of w. PP. across the 2.8 to 28 Mg capacity.

Economies of scale is a way to maximize production and minimize the cost of production in the business. Hence, economies of scale is the competitive advantage that large-scale production has over small-scale production. The larger the scale of production, the lower the per-unit costs, as the average costs can be spread over more production units [40]. There is an increase in total cost as output increases, but the average cost of producing each unit falls simultaneously. Economies of scale is a significant facet of effectiveness in production [41]. This is illustrated in the capital and operational costs for the m. silo + acc. and m. silo scenarios, despite having been very high and beyond the financial limits of many farmers, as the average cost decreased with an increased handling capacity. The greater the number of members constituting a farmer co-operative, the greater the chance of members increasing their handling capacity, which can substantially reduce the financial burdens. The importance of farmer co-operatives has been highlighted already, and it would be prudent for farmers to form co-operatives rather than operating individually. Moreover, due to financial constraints, individual farmers could explore the option of using PICS rather than w. PP. + Phos. or w. PP. Farmers could increase their production scale to benefit from economies of scale, and this could help farmers accrue considerable profits.

### 3.4. Breakeven

**Figure 13:**
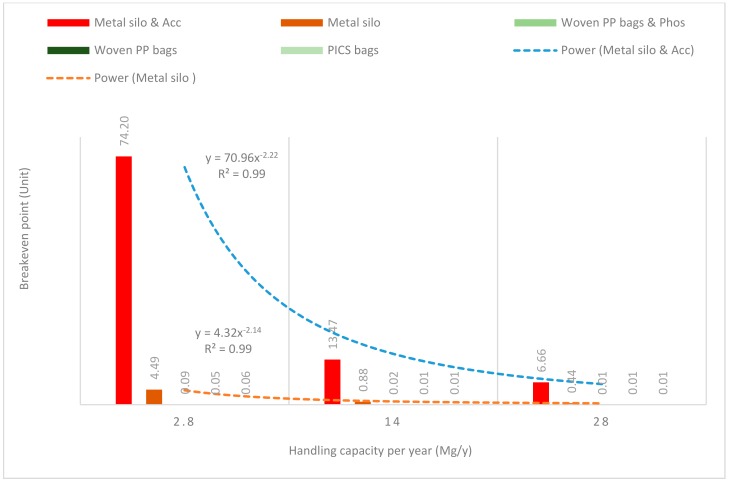
Breakeven results (the quantity of a particular handling capacity needed to breakeven) for each maize handling method per handling capacity/year.

Estimating the breakeven point is an essential step, and it has been considered in this study. A breakeven point is reached when the total revenue is equal to the total cost. Profit is zero at this point, but no money is lost. Revenue depends on the number of units sold. In general, revenue increases with an increasing number of units [42,43]. Figure 13 shows the breakeven points of handling maize grain using m. silo + acc., m. silo, w. PP. + Phos., w. PP., and PICS. In all these situations, the units (which are the quantity of a particular handling capacity needed to breakeven) for breakeven points decreased with the increased handling capacity. A drastic reduction from 74.20 units (at 2.8 Mg capacity) to 13.47 units (at 14 Mg capacity) was achieved handling grain with m. silo + acc. About 7 units (at 28 Mg capacity) were required to reach the breakeven point. When the grain was handled with m. silo, the breakeven point was reached at 14 Mg capacity. At 2.8 Mg capacity, only 4.5 units were required to breakeven. Of note, when grain was handled with w. PP. + acc., w. PP., and PICS, the breakeven point was attained at 2.8 Mg capacity.

The breakeven point was attained quickly using w. PP. + acc., w. PP., and PICS, even when the capacity was not increased. Hence, increasing capacity becomes an added advantage to farmers to make profits as quickly as possible. Continuous production capacity at 2.8 Mg/y or 28 Mg/y respectively demands 74 y or 7 y to reach the breakeven point. Quickly attaining the breakeven point requires an increase in the production unit per year, or an increase in the unit selling price, or a reduction if the average fixed and variable costs. According to [44], to reach a breakeven point, either the price of the product is increased or the fixed and variable costs are reduced. The breakeven points demanded higher production units using m. silo + acc., and m. silo because the fixed and variable costs were high compared to that of w. PP. + acc., w. PP., and PICS.

## 4. Conclusions

The annual capital cost and operational cost of handling maize grain with m. silo + acc., and m. silo were very high, and farmers could only afford the costs by forming farmer co-operatives. This could enhance their chances of accessing financial credits from banks, and also accumulate the required equity capital. Alternatively, the capital cost and operational cost of grain handling using PICS, w. PP. + Phos., and w. PP were essentially low and within the financial capabilities of individual smallholder farmers. We should not overlook the high losses in grain quality and quantity due to insects and pests, and the low quality and small quantity losses associated with grain handling using w. PP., and w. PP. + Phos. In this instance, grain handling using PICS is superior (high grain quality and quantity because it prevents the survival of stored-products insects) despite the relatively slight increase in capital cost. Nonetheless, it had the lowest operational cost. Mechanized handling of grain (m. silo + acc.), which has the ability to prevent insect, pest, and mold infestations, should have been the best option. This is because the grain quality and quantity are maintained, but due to financial constraints, farmers could opt for PICS bags on an individual basis.

The annual capital and operational costs decreased with the increase in production scale (economies of scale). It is significant that farmers increase their production capacity to make a considerable profit. Handling grain with m. silo + acc. or m. silo demanded the sale of many units (the quantity of a particular handling capacity needed to breakeven) of grain to reach the breakeven point. However, the number of units reduced as the production capacity was increased. On the contrary, to reach the breakeven point using m. silo, fewer production units were needed as the fixed and variable costs were lower than that of m. silo + acc. Even at the lowest capacity of 2.8 Mg, the breakeven point was attained using PICS, w. PP., and w. PP. + Phos. Predictably, increasing handling capacity increases the profit margin of farmers, however, the high percentage of grain weight loss mostly related to the use of w. PP., and w. PP. + Phos. should be not be disregarded. The high percentage of grain weight loss is mostly due to grain damage caused by stored-product insects. Food security and financial prospects of farmers can be high if maize grain is handled with m. silo + acc. or m. silo through farmer co-operatives. That notwithstanding, individual farmers could equally improve their food security and financial security by choosing PICS (or other airtight bags) rather than w. PP. or w. PP. + Phos.

The government of Ghana should initiate the application of m. silo + acc. in grain handling to complement the success of the many agricultural flagship programs.

## Figures and Tables

**Table 1:** Experimental design and parameters used for cost analysis.

Independent Variables	Scenarios
Grain handling and storage methods	1. Metal silo/thresher/gravity separator/screw conveyor/HDPE + UV solar tent dryer
	2. Metal silos/rubber
	3. Hermetic bag/rubber
	4. Insecticides (phosphine tablet)/polypropylene/rubber
	5. Polypropylene/rubber
Grain storage capacity in Mg	2.8
	14
	28
Estimated Losses in grain quantity/quality	0% to 7% grain loss for metal silo + acc., and metal silo only ^1^
	0% to 30% grain loss for woven PP only, woven PP + Phosphine, PICS bags only ^1^
Estimated price increase of grain	0%
	30%
	50%
	70%

^1^ Based upon [3,4,9,10,11,12,13].

**Table 2:** Specifications and prices of equipment and materials used for the cost analysis.

Equipment/Materials	Features	Average Unit Cost (US$)	Source
HDPE +UV Solar tent dryer (2 units bought)	(15 m × 3.6 m) × 2 = 108 m^2^, Lifespan: 25 y	1420.4	[14]
Thresher (1 unit bought)	9 Mg/hour, 4.5 kWh, Lifespan: 25 y	800.0	
Gravity separator (1 unit bought)	3.63 Mg/hour, 2.2 kWh, Lifespan: 25 y	14,550.0	
Screw conveyor (1 unit bought)	0.1 Mg/h, 1.5 kWh, up to 45° elbows, Lifespan: 25 y	1500.0	
Metal silo (1 unit bought)	28 Mg, Lifespan: 25 y, bottom hopper	3500.0	
Rubber	1 Mg/y, lifespan: 1 y	15.0	Personal inquiry
Woven polypropylene bags	1 Mg/y, lifespan: 1 y	10.4	
Phosphine tablets	1 Mg/y, Applied every 3 months/y	16.7	
Hermetic (PICS) bags	1 Mg/y, lifespan: 2–4 y (3 y used)	30.0	[15,16]

**Table 3:** Assumptions for utility, service, and miscellaneous prices used for the cost analysis.

Utility/Services	Estimates	Cost (US$ or %)	References
Electricity price (EP)	1 kWh	$0.06	[17]
Labor cost	Per day	$2.02	[18]
Operational hours of equipment (OH)	56 h/y or 2 h/day for 28 days	i.e., $14.14/y/person	Estimation
Number of hired laborers (Most activities to be carried out by farmers)	Two persons for using m. silo + acc., and 1 person for the others		Estimation
Interest rate (I)		17%	[19]
Monthly installment (N)	60 months		
Insurance rate	15–23%	10%	[20]
Tax (cooperate)		25%	[21]
Tax (individual)		7.5%	
Ghana cedi/dollar rate	GHȻ4.8	$1	[19]
Wiring & controls cost (C2)	Percentage of total capital cost of equipment (CP)	4%	[22]
Installation (C3)	Percentage of total capital cost of equipment (CP)	40%	
Motor efficiency		75%	
Equipment freight (C4)	Percentage of total capital cost of equipment (CP)	10%	Estimation

**Table 4:** Equations used for the cost analysis calculations.

Analyses (Units Included)	Equations (Units Included)
Grain handling capacity (g) in metric tons/day (Mg/d)	(1) Mg/d
Grain handling capacity (G) in metric tons/year (Mg/y)	(2) g (Mg/d) × OH (h/y)/(X h/d)
Annuity	(3) A = P (I (1 + I)^N^)/((1 + I)^N^− 1))
Equipment initial cost (CP), ($)	(4) Ʃ (purchase price of each piece of equipment)
Total equipment initial cost (C5), ($)	(5) Ʃ (CP + C2 + C3 + C4)
Total building cost (C6), ($)	(6) Building space (ft^2^) × 12.5 (Building construction cost, $/ft^2^)
Engineering and design cost (C7), ($)	(7) 7% of Ʃ (C5 + C6)
Electricity consumed (E1), motor load (kW-h/y)	(8) Connected load × OH/0.75
Total electricity cost (C8), ($/y)	(9) E1 × EP
Equipment salvage value (ESV), ($)	(10) 15% of Ʃ (C5 + C6)
Yearly sales of the product ($)	(11) The unit price of product × quantity on sale
Total Annualized Benefits (TAB), ($/y)	(12) (Annualize ESV + yearly sales of a product)
Annualized capital costs (AFC1), ($/y)	(13) Annualized (Ʃ(C5 + C6 + C7))
Annual straight-line depreciation (AFC2), ($/y)	(14) [(Cost basis of fixed asset − Salvage value)/(Estimated useful life)]
Insurance (AFC3), ($/y)	(15) 0.1 × (C5 + C6)
Interest (AFC4), ($/y)	(16) 0.17 × (C5 + C6)
Overhead (AFC5), ($/y)	(17) 0.16 ($/Mg) × G (Mg/y)
Taxes (AFC6), ($/y)	(18) Cooperate = 25/100 × (C5 + C6) Individual = 7.5/100 × (C5 + C6)
Total annual fixed costs (TAFC), ($/y)	(19) Ʃ (AFC1 + AFC2 + AFC3 + AFC4 + AFC5 + AFC6)
Electricity (AVC1), ($/y)	C8
Labor (AVC2), ($/y)	(20) 0.2525 ($/labor h) × 56 (operation h/y) × 2 (laborers)
Maintenance & repairs (AVC3), ($/y)	(21) 2 ($/Mg) × G (Mg/y)
Misc. supplies AVC4), ($/y)	(22) 1 ($/Mg) × G (Mg/y)
Other (AVC5), ($/y)	(23) 0.25 ($/Mg) × G (Mg/y)
Total annual variable costs (TAVC), ($/y)	(24) Ʃ (AVC1 + AVC2 + AVC3 + AVC4 + AVC5)
Total yearly Profit ($/y)	(25) TAB − (AFC1 + TAFC + TAVC)
Economies of scale ($/Mg/y)/Mg	(26) The capital or operational costs ($/Mg/y)/storage capacity (Mg)
Breakeven point (units)	(27) [(Annual fixed cost)/(Annual sale—Annual variable cost)]
